# Using fractional exhaled nitric oxide (FeNO) to diagnose steroid-responsive disease and guide asthma management in routine care

**DOI:** 10.1186/2045-7022-3-37

**Published:** 2013-11-07

**Authors:** David Price, Dermot Ryan, Annie Burden, Julie Von Ziegenweidt, Shuna Gould, Daryl Freeman, Kevin Gruffydd-Jones, Anne Copland, Clifford Godley, Alison Chisholm, Mike Thomas

**Affiliations:** 1 Research in Real Life, Cambridge, UK; 2 Respiratory Effectiveness Group, Cambridge, UK; 3 Division of Applied Health Sciences, University of Aberdeen, Aberdeen, UK; 4 Woodbrook Medical Centre, Loughborough and Honorary Fellow at the University of Edinburgh, Edinburgh, UK; 5 Mundesley Medical Practice and Norfolk Community Health & Care, Norfolk, UK; 6 Box Surgery, Wiltshire; Respiratory Lead, Royal College of General Practitioners, London, and Honorary Lecturer, University of Bath, Bath, UK; 7 Woodstock Medical Centre, Lanark, UK; 8 Avondale Medical Practice, Strathaven, UK; 9 Primary Care Research, University of Southampton, Southampton, UK

**Keywords:** Fractional exhaled nitric oxide (FeNO), Practical guidance, Diagnosis, On-going asthma management, Steroid-responsive disease

## Abstract

**Background:**

Fractional exhaled nitric oxide (FeNO) is a surrogate marker of eosinophilic airway inflammation and good predictor of corticosteroid response.

**Aim:**

To evaluate how FeNO is being used to guide primary care asthma management in the United Kingdom (UK) with a view to devising practical algorithms for the use of FeNO in the diagnosis of steroid-responsive disease and to guide on-going asthma management.

**Methods:**

Eligible patients (n = 678) were those in the Optimum Patient Care Research Database (OPCRD) aged 4–80 years who, at an index date, had their first FeNO assessment via NIOX MINO® or Flex®. Eligible practices were those using FeNO measurement in at least ten patients during the study period. Patients were characterized over a one-year baseline period immediately before the index date. Outcomes were evaluated in the year immediately following index date for two patient cohorts: (i) those in whom FeNO measurement was being used to identify steroid-responsive disease and (ii) those in whom FeNO monitoring was being used to guide on-going asthma management. Outcomes for cohort (i) were incidence of new ICS initiation at, or within the one-month following, their first FeNO measurement, and ICS dose during the outcome year. Outcomes for cohort (ii) were adherence, change in adherence (from baseline) and ICS dose.

**Outcomes:**

In cohort (i) (n = 304) the higher the FeNO category, the higher the percentage of patients that initiated ICS at, or in the one month immediately following, their first FeNO measurement: 82%, 46% and 26% of patients with high, intermediate and low FeNO, respectively. In cohort (ii) (n = 374) high FeNO levels were associated with poorer baseline adherence (p = 0.005) but greater improvement in adherence in the outcome year (p = 0.017). Across both cohorts, patients with high FeNO levels were associated with significantly higher ICS dosing (p < 0.001).

**Conclusions:**

In the UK, FeNO is being used in primary practice to guide ICS initiation and dosing decisions and to identify poor ICS adherence. Simple algorithms to guide clinicians in the practical use of FeNO could improved diagnostic accuracy and better tailored asthma regimens.

## Background

Inhaled anti-inflammatory (corticosteroid, ICS) therapy is the cornerstone of asthma management with the ultimate goal of maintaining control of the clinical manifestations of the disease for prolonged periods [1]. Initiation of maintenance therapy as either low-dose ICS or as a leukotriene receptor antagonist (LTRA) is recommended for patients who remain symptomatic despite as-needed short-acting bronchodilator therapy [2,3]. In patients who remain sub-optimally controlled on low-dose ICS, an increase in ICS dose (to medium- or high-dose), or the addition of a long-acting bronchodilator (LABA) or LTRA is recommended. For those who continue to be uncontrolled, further management options include higher doses of ICS, maintenance oral corticosteroids and anti-IgE therapy [2,3].

Achieving optimum asthma control requires an understanding of the nature and extent of the airway inflammation present. In the Gaining Optimum Asthma controL (GOAL) study, for example, over 30% of patients failed to achieve totally controlled asthma despite receiving maximum doses of fluticasone propionate. While drug delivery and/or treatment adherence may have played a part in their failure to achieve control, it may also have been partially attributable to the presence of steroid unresponsive asthma in these patients [4,5].

ICS dosing decisions (starting dose and subsequent dose adjustments) are typically symptom-driven and reliant on patient or carer/parental reports [6]. Yet respiratory symptoms can be non-specific and are not necessarily related to the extent or severity of the inflammation present [7]. Moreover, lung function and symptom scores have only a modest correlation with airway inflammation [8]. Indeed, although eosinophilic airway inflammation has been shown to be closely linked to steroid response [9-14], it has only a weak correlation to airway dysfunction and to symptoms [15,16]. Thus, a standard clinical assessment that relies on symptom assessment and simple lung-function tests alone provides limited information about the presence, or absence, of airway inflammation, or the extent to which a patient is at risk of future asthma exacerbations.

### Fractional exhaled nitric oxide

Approximately 50% of asthma cases are attributable to eosinophilic airway inflammation [17-19]. Eosinophilic airway inflammation results from the activation of mast cells and antigen-specific Th2 cells resulting in the production of cytokines and up-regulation of epithelial inducible nitric oxide (NO) [2]. The fraction of NO exhaled (FeNO) is elevated in patients with eosinophilic airway inflammation, yet generally remains low in patients with non-eosinophilic asthma [14]. FeNO has been shown to be closely correlated to findings using induced sputum and more invasive measures of airway inflammation (e.g. as bronchial biopsy). Thus, as a surrogate marker of eosinophilic airway inflammation and a good predictor of corticosteroid response (both positive and negative), FeNO has been proposed as a useful biomarker in patients with asthma [20-23].

The role of FeNO as a diagnostic and decision-support tool is relatively well documented [24-26]. Indeed, in 2011 the American Thoracic Society (ATS) published a comprehensive review of the literature in a clinical practice guideline that endorsed (strong recommendation; weak evidence) the use of FeNO as a quantitative, noninvasive, simple, and safe method of measuring airway inflammation and provides clinical guidance on its use for the diagnosis and management of asthma [24].

Yet despite the body of evidence underpinning the potential uses of FeNO, its uptake within primary care remains limited. This is, in part, due to a lack of clear, practical guidance on its appropriate use in routine asthma care and how it can be best utilized to aid diagnosis and support decision making with respect to ICS dose optimization and the need for non-ICS therapies.

Against this backdrop, we designed a study to identify patterns of use of FeNO assessment in routine primary care in the United Kingdom (UK) and the effects of this assessment on clinical parameters and physician and patient behaviour with a view to informing algorithms for the use of FeNO in primary care practice.

## Methods

### Data sources and patients

This observational study analyzed clinical data from the Optimum Patient Care Research Database (OPCRD). The OPCRD is a UK respiratory dataset containing anonymized, longitudinal, research-quality clinical records and patient-reported outcome data from practices that subscribe to the Optimum Patient Care (OPC) respiratory review service. At the time of the study, the OPCRD included 341,000 patients at 176 practices [27]. The OPCRD has been approved by Trent Multi Centre Research Ethics Committee for clinical research use, and the study protocol was approved by ADEPT (Anonymised Data Ethics Protocols and Transparency Committee), the OPCRD’s independent scientific advisory committee.

Eligible patients were those aged 4–80 years who, at an index date, had their first FeNO measurement via NIOX MINO® or Flex®. Patients had to be managed at practices routinely measuring FeNO, defined as those that measured FeNO in at least ten patients over the study period. All eligible patients had to have at least one (baseline) year of continuous practice data before the index date, and one complete (outcome) year of data after the index.

During baseline, all patients were characterised in terms of demography, clinical management, exacerbation history, risk and control status. Patients were defined as controlled in baseline if they used little symptom relief medication (average SABA use of ≤200 μg daily) and high risk if they had multiple (i.e. at least two) exacerbations over the baseline year. Normal, intermediate and high FeNO levels were categorised as: low/normal: <25 ppb (adults), <20 ppb (children); intermediate: 25–50 ppb (adults), 20–35 ppb (children); or high: >50 ppb (adults), >35 ppb (children) (see Table 1).

**Table 1:** Categorisation of FeNO level - low/normal; intermediate; high by age (i.e. adults and children)

	**Adults/Adolescents (Ages 12 and older)**	**Children (Ages 4–11)**
Low/normal (ppb)	<25	<20
Intermediate (ppb)	≥25 and ≤50	≥20 and ≤35
High (ppb)	>50	>35

Normal FeNO level = <25 ppb/<20 ppb in patients age ≥12/< 12 years. Intermediate FeNO level = 25–50 ppb/20-35 ppb in patients age ≥12/< 12 years. High FeNO level = >50 ppb/>35 ppb in patients age ≥12/< 12 years.

The implications of FeNO measurement and monitoring were evaluated in two patient cohorts: (i) those in whom FeNO measurement was being used to identify possible steroid-responsive disease (i.e. in patients not receiving ICS therapy during the baseline year) and (ii) those in whom FeNO monitoring was being use to support clinical management of asthma (i.e. in patients who received at least one prescription for ICS therapy during the baseline year).

### Study endpoints

In patients in whom FeNO measurement was being used to help identify possible steroid-responsive disease, the percentage of patients initiating ICS at, or in the one month immediately following their first FeNO measurement was evaluated and split by FeNO category (low/normal, intermediate, high) to explore the effect of FeNO measurement on physician prescribing behaviour.

In the cohort of patients in whom FeNO monitoring was being use to support asthma clinical management, the association between adherence to ICS in the baseline and outcome years (and change in ICS adherence between baseline and outcome) was evaluated, and split by FeNO category, to explore the effect of FeNO level on subsequent patient behaviour. Adherence was assessed in terms of medication possession ratio (MPR, defined as number of days’ supply of therapy/number of days in the total prescribing period × 100%).

Across both cohorts, the association between FeNO level and prescribed ICS dose was evaluated, with ICS dose being a marker of FeNO’s effect on physician prescribing behaviour.

### Statistical analyses

Analyses were conducted using SPSS version 18 (SPSS Statistics, IBM, Somers, NY, USA), SAS version 9.2 (SAS Institute, Marlow, Buckinghamshire, UK), and Microsoft Office Excel 2007 (Microsoft, Bellevue, WA, USA). Statistical significance was defined as p < 0.05. Descriptive analysis was used to investigate patterns of FeNO assessment and the effect on physician prescribing. Plots and statistical tests were used to explore the relationships between FeNO levels and ICS consumption/patient adherence using, namely Kruskal Wallis Test for variables measured on the interval/ratio scale and Chi Square Test for categorical variables.

## Results

### All patients

A total of 678 eligible patients were identified in the database. The median (interquartile range) age of the study population was 46 (26–60) years, 46% of patients were male, 45% had comorbid rhinitis and 6% of patients were current smokers. During baseline, 7% of the patients experienced multiple exacerbations, classifying them as high risk (12% had 1 exacerbation and 81% had no exacerbations during baseline) (see Table 2).

**Table 2:** Summary baseline characteristics of the eligible study population

**Baseline characteristics**			**Study population n = 678**
Sex: male n (%)			313 (46.2)
Age: median (IQR)			46 (26, 60)
BMI, median (IQR)			26.0 (22.9, 30.6)
Rhinitis diagnosis or therapy, n (%)			305 (45.0)
Smoking status, n (%)	Current Smoker		40 (5.9)
	Ex-smoker		145 (21.4)
	Non-smoker		477 (70.4)
Exacerbations, n (%)	0		551 (81.3)
	1		83 (12.2)
	≥2		44 (6.5)
SABA dose (mcg), n (%)	1-100		121 (17.8)
	101-200		132 (19.5)
	201-400		94 (13.9)
	401+		47 (6.9)
Average ICS Daily dose, (mcg) BDP-equivalent dose, n (%)	None		304 (44.8)
	1-100		67 (9.9)
	101-200		106 (15.6)
	201-400		101 (14.9)
	401+		100 (14.7)
Asthma therapy, n (%)	None		193 (28.5)
	SABA		101 (14.9)
	±SABA	LTRA	8 (1.2%)
		ICS	182 (26.8)
		ICS + LABA	136 (20.1)
		ICS + LAMA	1 (0.1)
	±SAMA	ICS + LABA + LAMA	5 (0.7)
		ICS + LTRA	8 (1.2)
		ICS + LABA + LTRA	42 (6.2)
	Other		2 (0.3)

Precise FeNO values were available for 73% of the population (n = 497) of whom, 56% had low/normal FeNO levels, 27% had intermediate levels and 17% had high levels. Across all patients, the higher the FeNO category, the higher the median prescribed ICS dose in the outcome year – a sign that FeNO measurement/monitoring was informing rationale prescribing decisions (see Table 3).

**Table 3:** Median ICS dose increase following initial FeNO reading, split by FeNO category

	**Initial FeNO reading**			**Total**	**p-value (Kruskal Wallis)**
	**Low**	**Intermediate**	**High**		
n (%)	276 (55.5)	135 (27.2)	86 (17.3)	497 (100.0)	<0.001
Median (IQR) ICS dose increase (μg)	0 (0–261)	152 (0–382)	219 (109–429)	-	

### Identifying possible steroid-responsive disease

**Figure 1:**
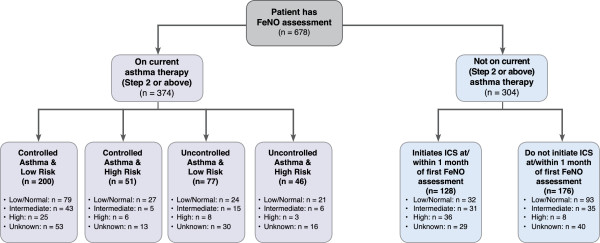
**Algorithm by showing how FeNO was used in 678 patients from the OPCRD.** Patients were aged ≥4 years with at least one year’s continuous patient records prior to their first FeNO reading.

FeNO measurement was being used to help identify possible steroid-responsive disease in 304 patients. ICS was initiated within one month of the index date (i.e. first FeNO measurement) in 82% of patients with high FeNO levels, 46% of those with intermediate FeNO levels and approximately one quarter (26%) of patients with low/normal FeNO (see Figure 1).

### Monitoring FeNO as part of asthma clinical management

**Figure 2:**
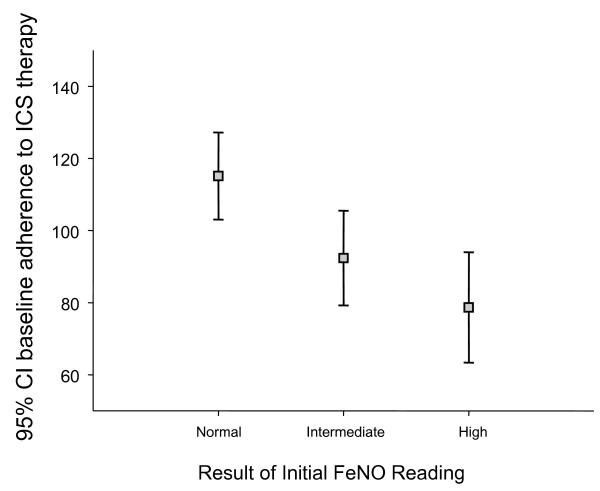
**Adherence to ICS during baseline year, split by FeNO category at time of initial FeNO measurement.** Legend: n = 260: Patients from OPCRD who were prescribed an ICS in year prior to 1st FeNO reading AND a recorded FeNO reading.

**Figure 3:**
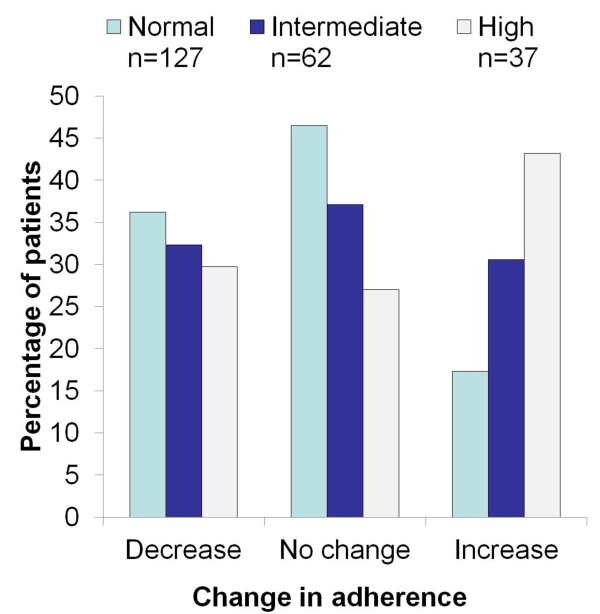
**Change in ICS adherence in the year following initial FeNO measurement, split by FeNO category.** Legend: n = 226: Patients from OPCRD who were prescribed ICS in the year prior to and post the 1st FeNO reading and in whom a specific FeNO value was recorded at time of first measurement.

Of the 678 total patient population, 374 had received at least one prescription for ICS (alone or in combination with other asthma therapy) prior to their first FeNO measurement (see Figure 1). There was no apparent relationship between baseline asthma control status or exacerbation rate and initial FeNO level, although only a relatively small percentage (16%) of patients had high FeNO at time of first assessment (see Figure 1). However, there was a significant association between baseline adherence and FeNO level at index date, with higher initial FeNO levels associated with lower baseline adherence (p = 0.005, see Figure 2). Higher FeNO levels were also associated with greater improvement in adherence in the following year – a proxy marker of patient reaction to FeNO monitoring (p = 0.017, see Figure 3).

## Discussion

The results of this real-life evaluation of FeNO monitoring in UK primary care practice suggest FeNO is being used (by the subgroup of practices included in the study) as a complementary diagnostic tool to aid diagnosis in patients in whom traditional assessment tools may have been inconclusive. It appears to be being used to guide decisions around ICS initiation or step up.

While FeNO can provide useful information about the nature and presence (or absence) of eosinophilic airway inflammation and about the likelihood (or not) of ICS responsiveness, it can also be used to identify poor adherence. These data suggest that a high FeNO level appears to drive improved ICS adherence in routine care. Using FeNO in this way has the potential to optimize existing therapy and minimize inappropriate use of ICS in patients unlikely to respond.

This real-life study adds to a rather limited evidence base on the role of FeNO monitoring in asthma management. At one point, the combined findings of a Cochrane systematic review of FeNO-based asthma management and a meta-analysis of studies evaluating the role of FeNO or sputum eosinophils in asthma management of patients concluded FeNO-guided asthma management could not be recommended (for adults or children) and that further studies were needed [28,29]. This conclusion was subsequently challenged on the basis that both the Cochrane review and the meta-analysis used the *number* of participants who had asthma exacerbations during follow-up as the primary endpoint, which does not take into consideration the fact that some patients may have multiple exacerbations during the course of a study. Indeed, the National Institute for Health (NIH) considers time to first asthma exacerbation and/or asthma exacerbation rates to be the most meaningful outcomes for assessing asthma exacerbations [30]. A re-analysis of the data from the Cochrane review and the meta-analysis [28-30] using asthma exacerbation rates as the endpoint (and using the standard Cochrane meta-analysis approach) found the *rate of exacerbations* was significantly lower in patients receiving a FeNO-based asthma management strategy, in both adults and children [31-35].

**Figure 4:**
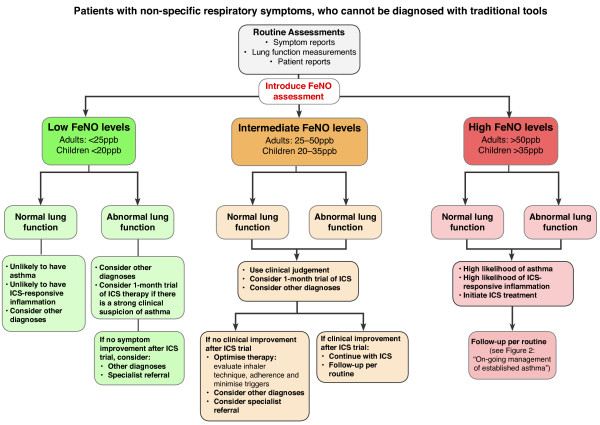
Clinical pathway for incorporating FeNO measurement at the time of initial evaluation of non-specific respiratory symptoms and diagnosis of steroid-responsive disease.

**Figure 5:**
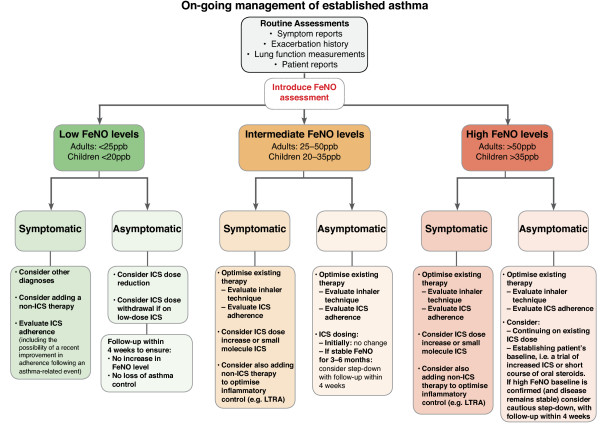
Clinical pathway for incorporating FeNO monitoring into on-going asthma management.

Pooling the evidence from the existing literature and the results of this real-life study together with our own experience of using FeNO in clinical practice, we have devised two clinical practice algorithms to aid primary care practitioners in the practical use of FeNO monitoring. The first algorithm is an “Initial Evaluation Algorithm”, which aims to provide guidance on the use of FeNO at the time of initial asthma diagnosis where traditional tools are inadequate (Figure 4). The second is an “On-going Monitoring and Decision Support Algorithm” to guide use of FeNO within longer-term asthma management and to inform prescribing decisions post diagnosis (Figure 5).

### Initial evaluation algorithms

The Initial Evaluation Algorithms build on an understanding that FeNO cannot be used to diagnose asthma (as asthma is defined by symptoms and variable airflow obstruction), but that it can be used to indicate elevated FeNO levels, the presence of eosinophilic airway inflammation and likely steroid response. Indeed, it can be a valuable complementary diagnostic tool and guide to therapeutic decision-making in patients with non-specific respiratory symptoms that are difficult to diagnose using conventional tools [9,10,24,25]. Where further investigations are felt necessary, we propose its inclusion in the diagnostic pathway after clinical evaluation and lung function testing. Our recommendations on how FeNO can be used at the time of initial evaluation of non-specific respiratory symptoms are summarized in Figure 4.

### Low FeNO levels

The presence of low FeNO levels in untreated patients with normal spirometry can help to rule out asthma (see Figure 4). Alternatively, low FeNO levels may indicate the possibility of non-eosinophilic asthma (or a diagnosis other than asthma). In either case, the likelihood of ICS-response is low and the likelihood of a diagnosis other than asthma is high. In general, ICS is not indicated in such patients, unless the clinical suspicion for asthma remains high (e.g. abnormal lung function, bronchodilator response), in which case a maximum 1-month trial of anti-inflammatory therapy could be an option before alternative diagnoses or specialist referral are considered.

### Intermediate FeNO levels

In isolation, the presence of intermediate FeNO levels in untreated patients with normal or abnormal spirometry does not confirm or exclude the presence of asthma. FeNO levels in the intermediate range, particularly when accompanied by evidence of airway obstruction, may indicate the possibility of eosinophilic asthma. However, FeNO levels in this range may also be consistent with non-eosinophilic asthma or a diagnosis other than asthma. If FeNO levels are found to be in the intermediate range and no definitive alternative diagnosis is evident, we recommend a 1-month trial of ICS (see Figure 4). If the patient responds to therapy (symptoms, lung function and/or normalization of FeNO), ICS should be continued and the patient monitored as per routine (including continued FeNO monitoring to help guide on-going management).

Where no improvement in FeNO and no objective clinical improvement has occurred following a therapeutic trial of ICS, we strongly recommend that alternative diagnoses or specialist referral be considered.

### High FeNO levels

High FeNO levels (with or without normal spirometry) indicate that eosinophilic airway inflammation is present and that the likelihood of ICS response is high. Consequently initiation of ICS therapy is recommended. However, specialist referral may still be necessary for symptomatic patients with lung function impairment and high FeNO levels if they do not respond to treatment with an ICS (see Figure 4). This scenario is discussed further below in the *On-going monitoring* section. In such patients it is particularly important to consider factors such as compliance with therapy, trigger factors, and inhaler technique before concluding a patient has not responded to therapy or that they need additional therapy.

### On-going monitoring and decision support algorithm

FeNO monitoring should be used in conjunction with structured assessments (validated assessment tools, lung function measurements, exacerbation history and patient reports) to provide supplementary information about a patient’s disease state and to help tailor asthma therapy appropriately. FeNO can be particularly useful when trying to identify patients who may be at increased risk of asthma exacerbations [36-38]; those who are non-compliant to corticosteroid therapy [39-41]; those who may be suitable for a change in therapy (step up or step down) [40,41], and when trying to assess whether corticosteroid-responsive airway inflammation has been controlled [42]. In addition, serial FeNO measurements can be used to monitor therapeutic response to (and adherence to) current therapy and to assess possible changes in allergen exposure in patients with known atopic inflammation.

Our recommendations on how FeNO can be used as a decision-support tool once a diagnosis of asthma has been established are summarised in Figure 5.

### Low FeNO levels

In the case of maintenance ICS patients who have low FeNO levels yet remain symptomatic, care must be taken to understand the reason for the persistence of symptoms. Alternative diagnoses and/or the presence of comorbidities (e.g. reflux, dysfunctional breathing) should be explored. Patients’ smoking habits should also be discussed. Not only can smoking reduce FeNO levels (possibly an early indicator of smoking’s impact on the lungs), but it can also drive management with higher ICS doses, both of which can increase the chance of a “false low” FeNO reading. Chronically reduced levels of FeNO have been demonstrated due to habitual smoking, but acute effects have also been shown immediately after cigarette smoking [42-46]. While the FeNO signal can be diminished by smoking, the depressant effect is not absolute and smokers with asthma can still have a raised FeNO [47]. To minimize the recording of falsely low FeNO reading, patients should be advised not to smoke in the hour before measurements, and short- and long-term active and passive smoking history should be recorded [48].

In patients with low FeNO levels in whom the diagnosis of asthma appears valid (e.g. confirmatory spirometry and bronchodilator response) increasing ICS is unlikely to result in an improvement in symptoms or other asthma outcomes. Instead, addition of a non-ICS therapy (e.g. an LTRA or LABA) should be considered. Also, as FeNO is highly ICS responsive (and low FeNO levels could indicate good adherence to ICS therapy) it is worth exploring whether there has been any recent change in a patient’s usual adherence pattern (e.g. an improvement in adherence in response to a recent exacerbation) that may have led to a short-term reduction in FeNO levels.

Where patients are asymptomatic, have low FeNO levels and have stable, well-controlled disease, a reduction in ICS dose (or ICS cessation if existing dose is low) should be considered. The chances of loss of control are much lower in patients with a low FeNO. Those selected for step-down should be followed-up (involving appropriate clinical investigations, including peak flow monitoring if appropriate) within 4 weeks, especially where the step-down is to low-dose ICS or where the ICS is being discontinued. If FeNO levels rise during the follow-up period, it may be a precursor to future loss of control and indicates the need to restart or increase ICS therapy. The 4-week follow-up recommendation is based on data from studies in both children and adults demonstrating that a 4-week period is adequate to identify individuals who are likely to experience an increase/decrease in their level of asthma control (based on increased/decreased FeNO levels) and be at increased/decreased risk of having an exacerbation [49-51]. A challenging group of patients to manage are those on high dose ICS (or high dose ICS containing regimens) who have some symptoms, but may not be particularly benefiting from high dose ICS. FeNO may be especially useful in guiding and reviewing step-down decisions in this group, but real-life research is required to evaluate the true value of FeNO in this particular clinical setting.

### Intermediate or high FeNO levels

We recommend no initial changes to existing ICS therapy, in asymptomatic patients with intermediate FeNO levels. Such patients should be followed for an additional 3–6 months and if they remain asymptomatic and their FeNO levels remain stable, a reduction in ICS dose can be considered with a follow-up in 4 weeks including an evaluation of symptom control, lung function and FeNO. However, it is important to recognize that in apparently stable patients, a rise in FeNO may indicate the possibility of a future loss of control. Thus, patients who have had an increase in FeNO levels to the intermediate zone, even in the absence of symptoms, should be followed more closely as they may be at risk of reduced asthma control. In all cases, medication adherence, trigger factors and inhaler technique should be carefully assessed.

On occasion, a patient with a high FeNO level and otherwise apparently well-controlled asthma and no history of frequent exacerbations will be encountered. The high but stable FeNO level may represent the “normal baseline” for some patients, but a trial of increased ICS (or even a 10–14 day course of oral corticosteroids) to definitively establish their “normal” baseline may be appropriate. Once a patient’s baseline has been established, and if they remain asymptomatic with stable FeNO levels, a reduction in ICS dose can be cautiously considered with follow-up in 4 weeks.

The presence of elevated (intermediate or high) FeNO levels in patients who remain symptomatic despite maintenance ICS therapy indicates sub-optimal control. For such patients, it is imperative to assess medication adherence, trigger factors and inhaler technique before any changes in treatment are considered. Where the patient remains symptomatic with elevated FeNO levels despite an evaluation and optimization of these factors, improved control may be possible by increasing the ICS dose or by addition of LTRA to improve anti-inflammatory management. Or specialist referral should be considered.

## Conclusions

In conclusion, FeNO monitoring should be viewed as a complementary assessment and monitoring tool when traditional assessment techniques prove inadequate or inconclusive. It can offer added advantages for clinicians (and patients) in terms of: (1) detecting the presence of eosinophilic airway inflammation, (2) determining the likelihood of ICS responsive (and lack thereof), (3) monitoring of airway inflammation to determine the potential need for a corticosteroid, and (4) unmasking (otherwise unsuspected) non-adherence to corticosteroid therapy.

Using FeNO to guide asthma management may not only benefit patients and reduce pressures on healthcare professionals, but could also reduce asthma-related healthcare costs [52-54]. FeNO-related cost savings could be realized through a variety of mechanisms, such as: reducing the use of inappropriately high doses of ICS (where ICS response has plateaued, or in patients who are stable and eligible for ICS dose reduction or cessation), and by reducing the cost of managing the effects of sub-optimal control (e.g. hospitalizations, exacerbations) in patients in whom guided step-up, or add-on therapy may be more beneficial. A UK cost-effectiveness study evaluated the relative comparative cost of using FeNO against other diagnostic and monitoring tools. For diagnosis, FeNO costs were compared against those for lung function and reversibility testing, bronchial provocation and sputum eosinophil count. For asthma management, the impact of FeNO monitoring on asthma control over one year (including ICS use, exacerbations and hospitalizations) was compared to symptoms and lung function (as in standard care). FeNO was measured using a hand-held monitor (NIOX MINO) at a reimbursement price of 23 pound sterling (£). The study found that an asthma diagnosis using FeNO cost £43 less per patient as compared with standard diagnostic tests. Asthma management using FeNO measurement (instead of lung function testing) resulted in annual cost-savings of £341 pound sterling for patients with mild to severe asthma and savings of £554 pound sterling for those with moderate to severe asthma [54]. While these data are of interest, further studies are required to explore the potential benefits and cost implications of FeNO-guided asthma management across a spectrum of asthma severities.

While some studies based in academic medical centres have found that physician-based treatment decisions are non-inferior to biomarker- or symptom-based ICS adjustments in mild-moderate asthma, [55] we believe that FeNO monitoring (use as discussed in our proposed clinical pathways) can assist primary care physicians who are not experts in respiratory disease to achieve improved diagnostic accuracy in patients with non-specific respiratory symptoms and to achieve more tailored and targeted treatment regimens for on-going asthma management.

## Competing interests

DP has consultant arrangements with Almirral, AstraZeneca, Boehringer Ingelheim, Chiesi, GlaxoSmithKline, Merck, Mundipharma, Medapharma, Novartis, Napp, Nycomed, Pfizer, Sandoz, Takeda and Teva. He or his research team have received grants and support for research in respiratory disease from the following organisations in the last 5 years: UK National Health Service, Aerocrine, AstraZeneca, Boehringer Ingelheim, Chiesi, GlaxoSmithKline, Merck, Mundipharma, Novartis, Nycomed, Orion, Pfizer, and Teva. He has spoken for Almirral, AstraZeneca, Activaero, Boehringer Ingelheim, Chiesi, Cipla, GlaxoSmithKline, Kyorin, Novartis, Merck, Mundipharma, Pfizer and Teva. He has shares in AKL Ltd which produces phytopharmaceuticals. He is the sole owner of Research in Real Life Ltd (RiRL) and its subsidiary social enterprise Optimum Patient Care. MT spoke for Aerocrine at the 2012 European Respiratory Society Annual Conference, he has no other conflicts of interest to declare. CG has participated in respiratory and primary care advisory and educational events organised by GlaxoSmithKline, Almirall and AstraZeneca and has received support from Aerocrine for Niox Mino mouthpiece provision. A Copland has spoken for Almirral, AstraZeneca, Boehringer Ingelheim, Chiesi, Glaxo Smith Kline, Merck Novartis and Pfizer. DF has spoken for Napp, Chiesi and Boehringer Ingelheim. At the time the data and opinions in this paper were analysed and formulated: AB, SG and JvZ are employees of RiRL; A Chisholm is a consultant to RiRL. DR and K G-J have with no conflicts of interests to declare.

## Authors’ contributions

DP, MT, DR, K G-J, A Copland, CG, conceived and designed the study. AB conducted the analyses, with help from JvZ and SH. A Chisholm wrote the initial draft of the paper. All authors contributed to the data collection and/or interpretation, the writing of the manuscript, and review of the final draft. DP is the study guarantor. All authors read and approved the final manuscript.
